# Computer aided quantification of intratumoral stroma yields an independent prognosticator in rectal cancer

**DOI:** 10.1007/s13402-019-00429-z

**Published:** 2019-03-01

**Authors:** Oscar G. F. Geessink, Alexi Baidoshvili, Joost M. Klaase, Babak Ehteshami Bejnordi, Geert J. S. Litjens, Gabi W. van Pelt, Wilma E. Mesker, Iris D. Nagtegaal, Francesco Ciompi, Jeroen A. W. M. van der Laak

**Affiliations:** 1 Department of Pathology, Radboud Institute for Health Sciences, Radboud University Medical Center, P.O.Box 9101, 6500 HB Nijmegen, The Netherlands; 2 Diagnostic Image Analysis Group (DIAG), Radboud University Medical Center, Nijmegen, The Netherlands; 3 Laboratory for Pathology East Netherlands (LabPON), Hengelo, The Netherlands; 4 Department of Surgery, Medisch Spectrum Twente, Enschede, The Netherlands; 5 Department of Surgery, Leiden University Medical Center, Leiden, The Netherlands; 6 Center for Medical Image Science and Visualization, Linköping University, Linköping, Sweden

**Keywords:** Rectal carcinoma, Tumor-stroma ratio, Prognosis, Computational pathology, Automated analysis, Deep learning

## Abstract

**Purpose:**

Tumor-stroma ratio (TSR) serves as an independent prognostic factor in colorectal cancer and other solid malignancies. The recent introduction of digital pathology in routine tissue diagnostics holds opportunities for automated TSR analysis. We investigated the potential of computer-aided quantification of intratumoral stroma in rectal cancer whole-slide images.

**Methods:**

Histological slides from 129 rectal adenocarcinoma patients were analyzed by two experts who selected a suitable stroma hot-spot and visually assessed TSR. A semi-automatic method based on deep learning was trained to segment all relevant tissue types in rectal cancer histology and subsequently applied to the hot-spots provided by the experts. Patients were assigned to a ‘stroma-high’ or ‘stroma-low’ group by both TSR methods (visual and automated). This allowed for prognostic comparison between the two methods in terms of disease-specific and disease-free survival times.

**Results:**

With stroma-low as baseline, automated TSR was found to be prognostic independent of age, gender, pT-stage, lymph node status, tumor grade, and whether adjuvant therapy was given, both for disease-specific survival (hazard ratio = 2.48 (95% confidence interval 1.29–4.78)) and for disease-free survival (hazard ratio = 2.05 (95% confidence interval 1.11–3.78)). Visually assessed TSR did not serve as an independent prognostic factor in multivariate analysis.

**Conclusions:**

This work shows that TSR is an independent prognosticator in rectal cancer when assessed automatically in user-provided stroma hot-spots. The deep learning-based technology presented here may be a significant aid to pathologists in routine diagnostics.

## Introduction

In most solid malignancies, therapeutic decision making is primarily based on pathological staging of tumors. The traditional tumor, (lymph) node, metastasis (TNM) staging system [1] is routinely used to estimate patient prognosis and guide treatment worldwide. For certain tumor types, however, the TNM system lacks accuracy in assessing the metastatic potential of a tumor. For instance, TNM stage II colorectal cancer (CRC) comprises a heterogeneous group with a diverse outcome [2]. As a result, the TNM stage is not informative for therapy planning of these patients, leading to both under- and over-treatment. Reliable new biomarkers are needed to guide personalized adjuvant treatment for these groups of patients.

A widely studied prognostic factor is the tumor-stroma ratio (TSR), expressing the relative amounts of tumor and intratumoral stroma. TSR is a straightforward measure which can be assessed by microscopic inspection of hematoxylin and eosin (H&E) stained tissue sections. TSR has been shown to yield prognostic information in a range of solid malignancies, including breast cancer [3–5] and lung cancer [6, 7]. Generally, TSR is an independent prognostic factor, where a high content of intratumoral stroma is associated with a poor prognosis. A number of previous studies showed promising results on the prognostic relevance of TSR in CRC [8–12]. Despite this evidence, there is no implementation in routine pathology reporting. This may be attributed to the variety in methodology and the lack of a standardized procedure for TSR assessment. Published studies propose visual assessment (‘eyeballing’), systematic point counting, and the use of scanned (digitized) tissue sections (whole slide images; WSI). Although good inter-observer agreement was found in earlier studies [9, 11, 13], visual assessment of pathological quantitative features in general may suffer from reproducibility issues.

To facilitate an objective and standardized TSR assessment, image analysis and machine learning algorithms have been applied on H&E-stained sections of CRC before, however, these algorithms were applied to image regions extracted from WSI. Computer-aided tumor and stroma quantification has been proposed based on automated tissue segmentation in H&E-stained sections using a combination of hand-crafted features and machine learning [14]. Furthermore, TSR has been computed via automated point counting in H&E-stained images [15]. Similar image analysis techniques based on classical machine learning have been applied to tissue microarrays for epidermal growth factor receptor (EGFR) detection by immunohistochemistry [16, 17]. A new branch of machine learning algorithms, so-called deep learning algorithms, have recently entered the field of computational pathology and shown promise for automating certain tasks in histopathology. Detection of sentinel lymph node metastases [18] and of cancer in prostate biopsies [19] could successfully be performed using convolutional neural networks (CNN), a specific type of deep learning. We recently showed [20] that a deep learning-based algorithm can distinguish between 9 different types of tissue in CRC WSI with an overall accuracy of 93.8%.

The present study aims to leverage our previously developed CNN for automated TSR assessment in the CRC sub-class of rectal adenocarcinomas. Only a limited number of studies have been published on TSR for rectal cancers and in a sub-analysis (*n* = 43) by West et al. [12] its prognostic value could not be confirmed. Work by Scheer et al. [8] recently showed that TSR has potential as a prognostic factor for survival in surgically treated rectal cancer patients, however, TSR was only found to be an independent prognosticator in lymph node metastasis negative cases. The performance of the automated TSR system described here will be compared with data from human experts and its prognostic value will be evaluated in terms of disease-specific and disease-free survival times.

## Materials and methods

### Patients

An existing cohort of 154 patients [8] with rectal adenocarcinoma stages I-III was used. All patients received curative surgery in the period 1996–2006 at the Medisch Spectrum Twente hospital (The Netherlands). No patient was neoadjuvantly treated with radiotherapy and/or chemotherapy or died within 30 days after surgery. At the time of surgery, none of the patients had known distant metastases, inflammatory bowel disease, hereditary nonpolyposis colorectal cancer (HNPCC) or other/earlier cancers. Histopathological data were obtained from the Laboratory for Pathology Eastern Netherlands (LabPON). Clinical data were obtained from the Medisch Spectrum Twente hospital and the Netherlands Comprehensive Cancer Organization (IKNL). Collected clinicopathological data included tumor grade (differentiation), depth of invasion (pT) and lymph node involvement (pN) according to the Union Internationale Contre le Cancer/American Joint Cancer Committee (UICC/AJCC) TNM staging system [1]. Data regarding adjuvant therapy and local or distant recurrence were also available.

### Tissue slide preparation and scanning

According to standard procedures at LabPON, formalin fixed and paraffin embedded tissue sections were cut at 2 μm and stained in an automatic stainer with hematoxylin and eosin (H&E) for routine diagnostic purposes. For the present study, a single slide per patient was selected which contained the most invasive part of the tumor and was used in diagnostics to assess the tumor pT-status. Slides were scanned at ×200 total magnification (tissue level pixel size ~0.455 μm/pixel) using a Hamamatsu NanoZoomer 2.0-HT (C9600–13) scanner (Herrsching, Germany).

### Visual estimation of intratumoral stroma

Two observers (GvP, WM; both > 10 years of experience with TSR scoring) independently scored the slides using a conventional light microscope according to a previously published protocol for TSR assessment [9, 10]. Briefly, the procedure consisted of 1) coarse localization of the tissue area with the highest intratumoral stroma content at low microscope magnification, and 2) selection of one field of view at ×100 total magnification and visual estimation of the tumor-stroma ratio (TSR-visual) in the selected circular region. Ideally, the selected region should meet the following criteria: high intratumoral stroma content (predominantly found at the invasive margin of a tumor); presence of tumor cells at all borders of the field of view; no large quantities of muscle, mucus, necrosis or large vessels; and no tears or tissue retraction artefacts. As much as possible, the region with the highest stroma content (stroma hot-spot) was selected that met all the above requirements. TSR-visual was estimated by both observers independently, using 10% increments. As a result of the specific microscope and lenses used, the specimen-level diameter of the circular region was 1.8 mm at ×100 magnification. There is a lot of variation among published studies concerning used TSR procedures (e.g. major differences in the location and size of the assessed tissue regions as well as what was actually measured: relative tumor or stroma content). For clarity, in this study the tumor-stroma ratio was defined as TSR = 100% × [intratumoral stroma area] / [tumor area + intratumoral stroma area]. Lumen, tears and other tissue types in the selected circular region were excluded during visual estimation. Lastly, the tissue region considered most suitable for TSR assessment was identified during a consensus meeting between the two observers in which 1) a binary TSR consensus score was determined: ‘stroma-low’ or ‘stroma-high’, and 2), the center of the stroma hot-spot was marked on the glass slide.

### Automated computation of intratumoral stroma

To study the value of applying a deep learning algorithm for automated TSR assessment (TSR-auto), a CNN was developed similar to a previously published algorithm [20]. The CNN performs tissue segmentation (i.e. subdivision of tissue areas) of H&E-stained rectal cancer WSI into nine different classes: tumor, intratumoral stroma, necrosis, muscle, healthy epithelium, fatty tissue, lymphocytes, mucus and erythrocytes. The CNN was trained using manually annotated regions in 74 WSI taken from the cohort used in this study. Regions to annotate were selected for covering tissue variety across WSI, rather than producing exhaustive annotations on a small number of WSI. Annotations were produced by a pathology researcher (OG) and a medical student, and were checked and corrected when deemed necessary by an experienced pathologist (AB). A digital staining normalization method [21] was applied to all WSI as a pre-processing step to accommodate for typical differences in tissue staining intensities, caused by variations in slide preparation. Unlike Ciompi et al. [20], here we used patches of 256 × 256 pixels for classification, which experimentally showed to improve performance and produce a smoother segmentation map (data not shown). Performance of the system was assessed by segmenting all WSI in the dataset in a five-fold cross validation fashion (at WSI level) and evaluating accuracy in all annotated regions.

**Fig. 1:**
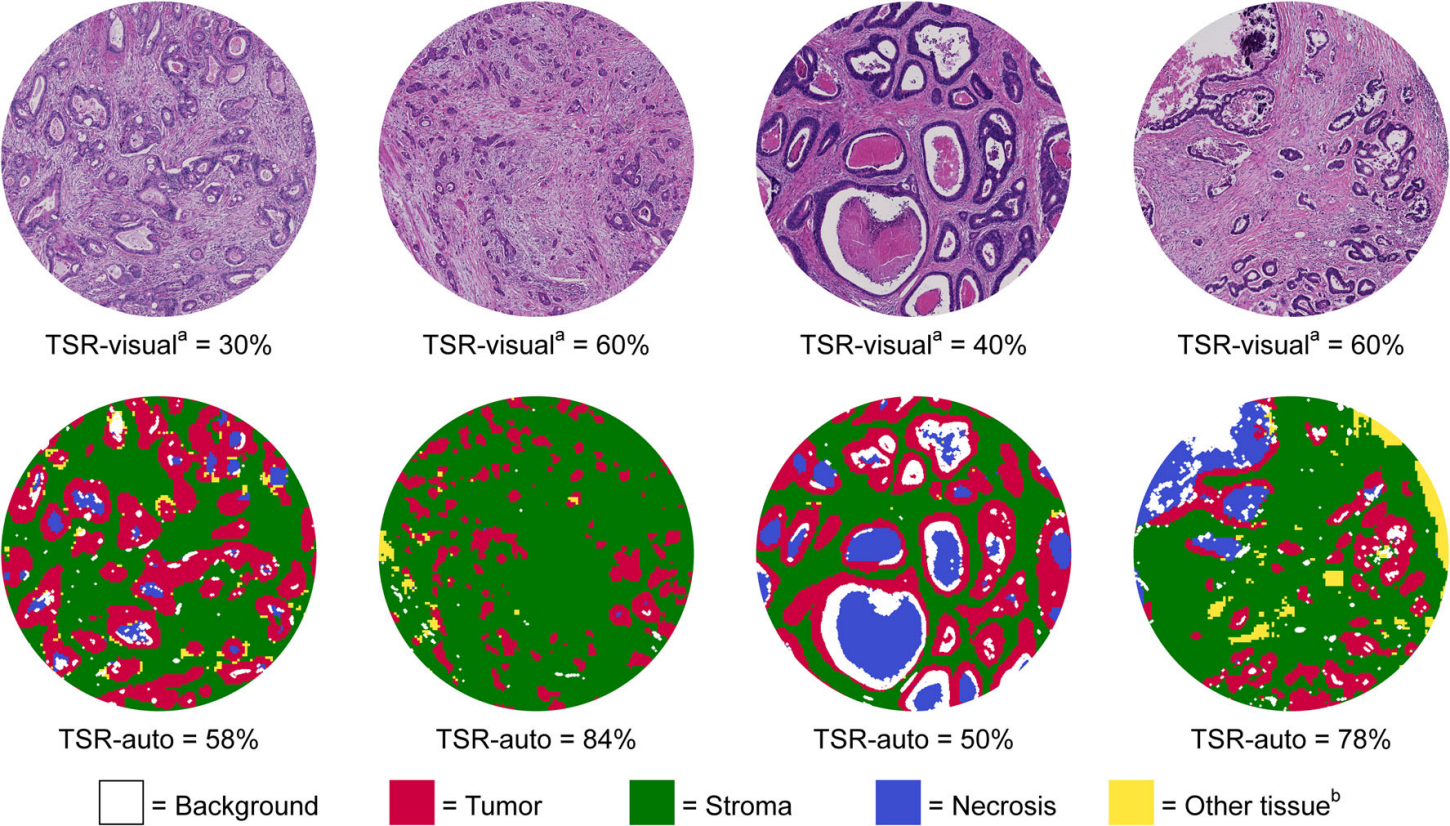
Top row: Stroma hot-spot circles, 1.8 mm across, selected by the observers for the assessment of TSR-visual^a^ and extracted with a diameter of ~4000 pixels for processing by the CNN. Bottom row: The same regions with tissues segmented by the CNN for the calculation of TSR-auto. ^a^Observer consensus; ^b^Other tissue includes classes: muscle, healthy epithelium, fatty tissue, lymphocytes, mucus and erythrocytes

To enable comparison, the CNN-based TSR-auto was computed in the same circular region (with 1.8 mm diameter) that was selected by the observers at the consensus meeting, where TSR-visual was assessed. The corresponding image data were extracted from each WSI as circles with a diameter of ~4000 pixels and processed further by the CNN described above (Fig. 1). Segmentation of a WSI into nine different tissue classes enabled in- and exclusion of specific tissue types comparable to the visual assessment procedure. The used definition of TSR-auto is similar to TSR-visual, expressing the area consisting of stroma as a percentage of the area occupied by both tumor and stroma.

### Statistical analyses

In this study, TSR-visual and TSR-auto were compared as prognostic factors in rectal cancer. Statistical analyses were performed using IBM SPSS software v24.0 (Armonk, NY, USA). The intraclass correlation coefficient (ICC) was used to determine the correlation between TSR assessed by two observers and by the automated method. To investigate a possible relationship between clinicopathological variables and the numerical values of TSR-visual and TSR-auto, Mann–Whitney U and Kruskal–Wallis tests were performed for two- and multi-class variables, respectively. For further statistical analysis, TSR-visual and TSR-auto were dichotomized, subdividing patients into two groups: ‘stroma-low’ and ‘stroma-high’. Dichotomization of TSR-visual was performed based on a cut-off value previously established [10] on 63 colon cancer cases: stroma-high = TSR-visual > 50% and stroma-low = TSR-visual ≤ 50%. In this study, we analyzed results for two different cut-off values for TSR-auto since the optimal cut-off value for the automated approach is not yet established. One method of dichotomization used the ‘50% stroma cut-off’, similar to TSR-visual, referred to as TSR-auto(50%), and the other dichotomization method was based on the median value for all measured TSR-auto values, referred to as TSR-auto(median), yielding equal numbers of patients in stroma-low and stroma-high groups.

Inter-observer agreements were calculated using Cohen’s Kappa (**κ**) on the dichotomized TSR values. Kaplan-Meier survival analyses were performed and log-rank statistics were used to test differences in both disease-specific survival (DSS) and disease-free survival (DFS) distributions. DSS was defined as the time between the date of surgery and the date of death attributable to rectal adenocarcinoma. For DFS, the date of the first event of cancer recurrence was used, which could be loco-regional or a distant metastasis. In case no event occurred, the time period until the last date of follow-up was used in the survival analyses. Finally, both uni- and multivariate analyses were performed for TSR-visual and TSR-auto using the Cox proportional hazards model. Probability values < 0.05 (2-sided) were considered statistically significant.

## Results

### Clinicopathological data

Of 154 cases projected for inclusion in this study, twelve cases with mucinous carcinoma were excluded as these tumors exhibit largely different TSR values. Twelve other cases were excluded because, at the time of writing, the required slides or data were unavailable. One case was excluded because the corresponding tissue slide did not contain invasive carcinoma.

The median follow-up time for the remaining 129 patients used in the present study was 5.6 years (interquartile range 2.3–8.3). The median age of the patients at the time of surgery was 67 years (interquartile range 59–74). Further clinicopathological data can be found in Table 1. There was no significant correlation between the clinicopathological variables and assessed values of TSR-visual or TSR-auto (*p* > 0.05).

**Table 1:** Clinicopathological data for 129 rectal cancer patients in relation to TSR-visual^a^ and TSR-auto

	Total	TSR-visual^a^		TSR-auto(50%)		TSR-auto(median)	
		Stroma-low	Stroma-high	Stroma-low	Stroma-high	Stroma-low	Stroma-high
	*n* (%)	*n* (%)	*n* (%)	*n* (%)	*n* (%)	*n* (%)	*n* (%)
Gender							
Female	43 (34)	30 (34)	13 (31)	11 (35)	32 (33)	22 (34)	21 (32)
Male	86 (67)	57 (66)	29 (69)	20 (65)	66 (67)	42 (66)	44 (68)
T-status							
pT1	4 (3)	4 (5)	0 (0)	2 (6)	2 (2)	3 (5)	1 (2)
pT2	40 (31)	29 (33)	11 (26)	5 (16)	35 (36)	18 (28)	22 (34)
pT3	79 (61)	51 (59)	28 (67)	24 (77)	55 (56)	42 (66)	37 (57)
pT4	6 (5)	3 (3)	3 (7)	0 (0)	6 (6)	1 (2)	5 (8)
N-status							
pN0	78 (60)	54 (62)	24 (57)	22 (71)	56 (57)	43 (67)	35 (54)
pN1	33 (26)	23 (26)	10 (24)	6 (19)	27 (28)	13 (20)	20 (31)
pN2	18 (14)	10 (11)	8 (19)	3 (10)	15 (15)	8 (13)	10 (15)
Stage							
I	33 (26)	26 (30)	7 (17)	6 (19)	27 (28)	18 (28)	15 (23)
II	45 (35)	28 (32)	17 (40)	16 (52)	29 (30)	25 (39)	20 (31)
III	51 (40)	33 (38)	18 (43)	9 (29)	42 (43)	21 (33)	30 (46)
Tumor grade							
Well	3 (2)	2 (2)	1 (2)	1 (3)	2 (2)	1 (2)	2 (3)
Moderate	112 (87)	73 (84)	39 (93)	28 (90)	84 (86)	55 (86)	57 (88)
Poor	14 (11)	12 (14)	2 (5)	2 (6)	12 (12)	8 (13)	6 (9)
Surgery type							
APR	62 (48)	39 (45)	23 (55)	10 (32)	52 (53)	26 (41)	36 (55)
LAR	49 (38)	37 (43)	12 (29)	17 (55)	32 (33)	29 (45)	20 (31)
Hartmann	18 (14)	11 (13)	7 (17)	4 (13)	14 (14)	9 (14)	9 (14)
Adjuvant treatment							
None	86 (67)	59 (68)	27 (64)	24 (77)	62 (63)	45 (70)	41 (63)
Radiotherapy	43 (33)	28 (32)	15 (36)	7 (23)	36 (37)	19 (30)	24 (37)
Chemoradioth.^b^	5 (4)	3 (3)	2 (5)	1 (3)	4 (4)	2 (3)	3 (5)

Mann–Whitney U and Kruskal–Wallis tests showed no significant correlation (*p* > 0.05) between the listed variables and TSR-visual or TSR-auto

LAR: Low anterior resection; APR: Abdominoperineal resection; pT: Pathological tumor status; pN: Pathological nodal status

^a^Observer consensus

^b^Chemoradiotherapy

### Performance of the deep learning system

Measures of sensitivity and specificity per tissue type as well as overall accuracy were assessed for the automatic method by pixel-wise comparison of predicted labels with ground truth labels in manually annotated regions. We found that the overall accuracy was 94.6%, which shows improvement on what was reported by Ciompi et al. [20]. Values of per-class sensitivity and specificity are reported in Table 2.

**Table 2:** Quantitative performance of the CNN at pixel classification per tissue class

	Tumor	Stroma	Necrosis	Muscle	Healthy epi.	Fat	Mucus	Lympho-cytes	Blood
Sensitivity	91.1%	91.7%	90.8%	95.5%	94.1%	98.1%	96.4%	98.4%	97.9%
Specificity	99.4%	97.7%	99.6%	99.6%	99.5%	99.9%	98.7%	99.6%	99.9%

CNN: Convolutional Neural Network; Healthy epi.: Healthy epithelium

Examples of tissue segmentation by the CNN in four circular regions selected by the observers are shown in Fig. 1. In line with the high classification accuracy, good segmentation of tumor, stroma and other tissues types was observed. Further qualitative inspection of the circular regions revealed some minor segmentation errors. Directly at the stroma-tumor interface, a very thin band of stroma pixels is often misclassified as tumor. Likewise, however, small groups of tumor cells (e.g. tumor buds, or thin tumor structures) were sometimes misclassified as stroma.

### Inter-observer and computer-observer agreement

**Fig. 2:**
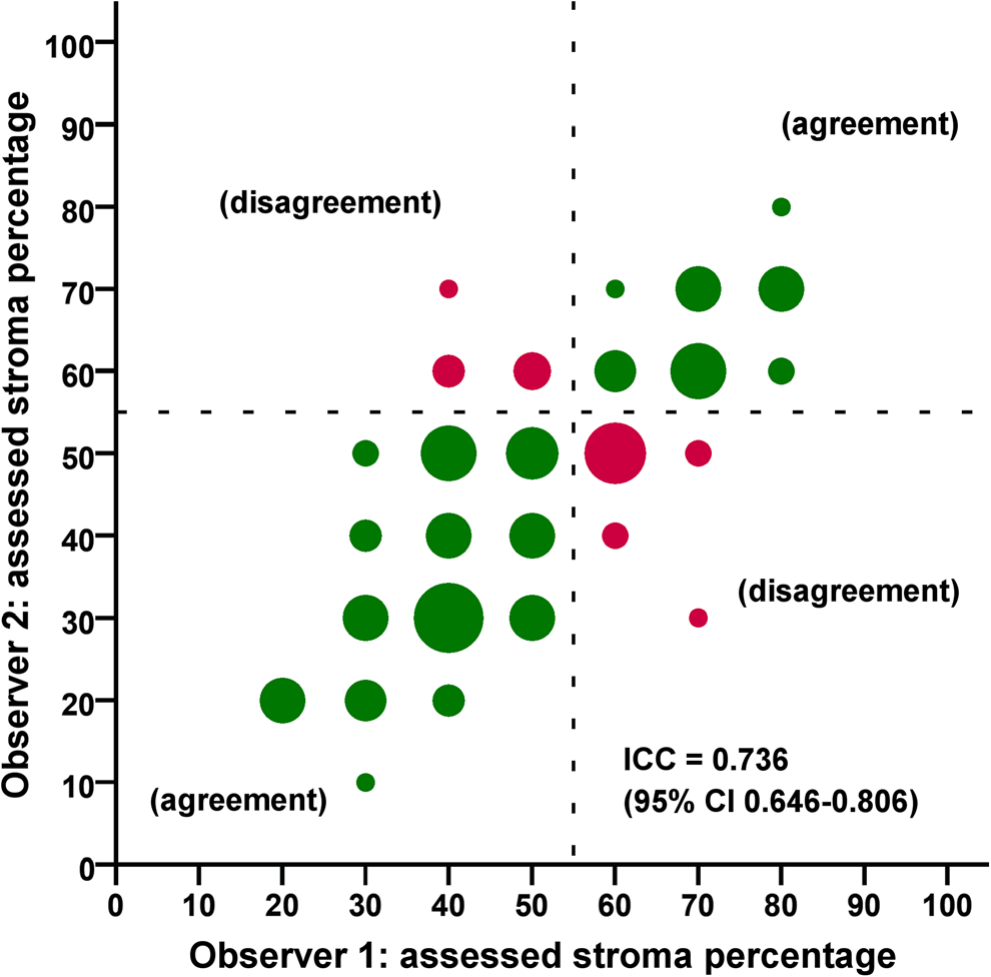
Scatter plot of assessed stroma percentages in 129 patients for Observer 1 and Observer 2. The co-occurrence of assessed percentages is indicated by circles with areas proportional to the amount of patients scored with the corresponding TSR value. The dashed lines represent the boundary between stroma-low and stroma-high cases according to the cut-off value determined in Mesker et al. [10]. Green circles indicate cases where the observers agreed (105 in total) and red circles indicate disagreement (24 in total)

The ICC between the two observers for the assessment of TSR was 0.736 (95% confidence interval (95% CI) 0.646–0.806). The co-occurrence of TSR scores assessed by the two observers is depicted in Fig. 2. The ICC’s between TSR-auto and TSR-visual were 0.475 (95% CI 0.330–0.598) and 0.411 (95% CI 0.257–0.545) for observers 1 and 2, respectively.

A moderate agreement between the two observers (**κ** = 0.578) was found after dichotomizing TSR-visual on basis of the 50% cut-off as described in section 2.5. Using the identical cut-off for TSR-auto, we observed only a fair agreement between TSR-visual and TSR-auto (**κ** = 0.239). Agreement improved considerably (**κ** = 0.521) when the median was used as cut-off for TSR-auto, resulting in: stroma-low = TSR-auto ≤ 65.47% and stroma-high = TSR-auto > 65.47%. Patients assigned to stroma-low or stroma-high groups by the observers and the automatic method are detailed in Tables 3, 4 and 5.

**Table 3:** Cross-tabulation of Observer 1 versus Observer 2 after dichotomisation

	**κ** = 0.578	Observer 2		
		Stroma-low	Stroma-high	Total
Observer 1	Stroma-low	75	8	83
	Stroma-high	16	30	46
	Total	91	38	129

**Table 4:** Cross-tabulation of TSR-visual (consensus) versus TSR-auto(50%) after dichotomisation

	**κ** = 0.239	TSR-auto(50%)		
		Stroma-low	Stroma-high	Total
TSR-visual (consensus)	Stroma-low	30	57	87
	Stroma-high	1	41	42
	Total	31	98	129

**Table 5:** Cross-tabulation of TSR-visual (consensus) versus TSR-auto(median) after dichotomisation

	**κ** = 0.521	TSR-auto(median)		
		Stroma-low	Stroma-high	Total
TSR-visual (consensus)	Stroma-low	60	27	87
	Stroma-high	4	38	42
	Total	64	65	129

### Survival analyses

**Fig. 3:**
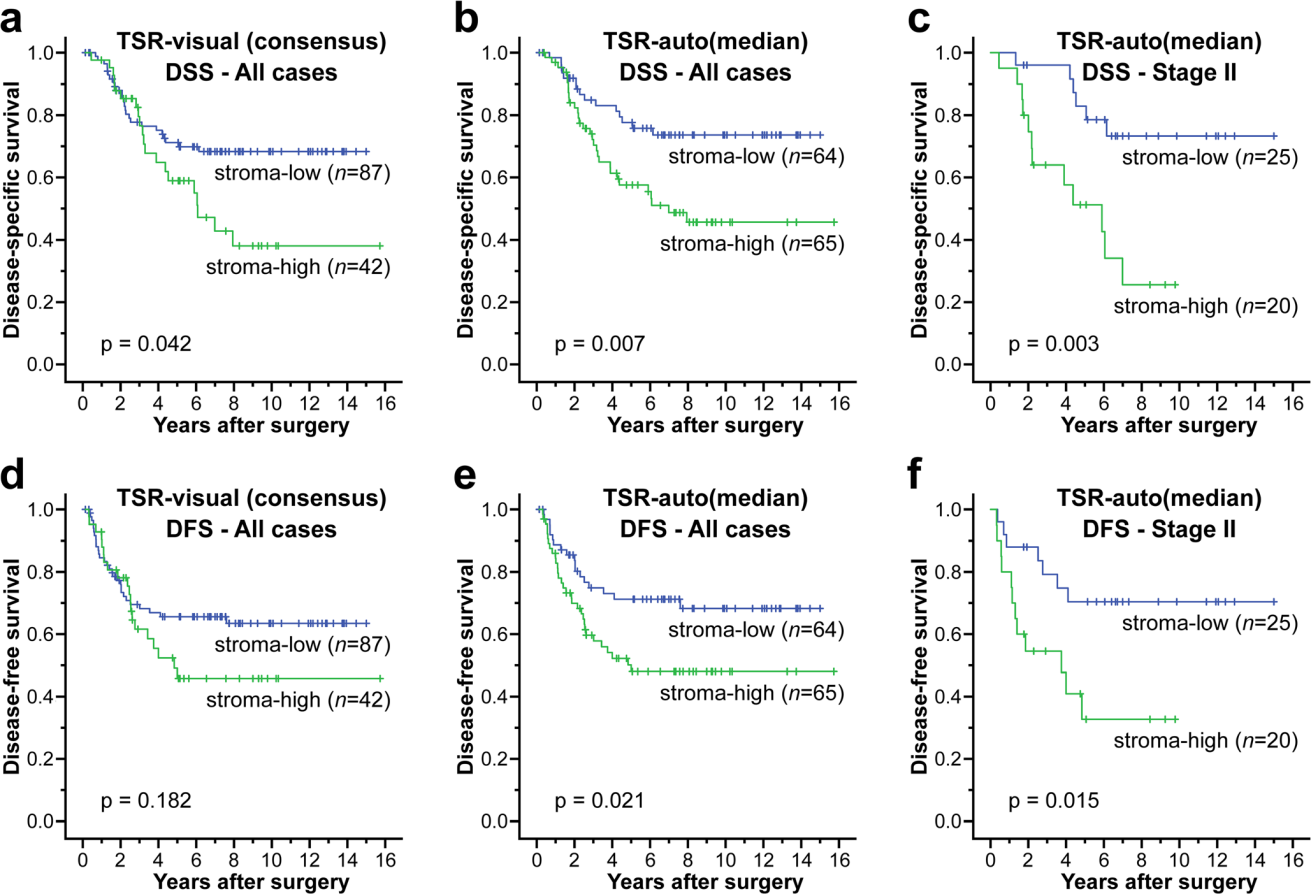
Kaplan-Meier curves for disease-specific survival (top row) and disease-free survival (bottom row) of stroma-low versus stroma-high patients. Results based on all patients (*n* = 129) are shown for TSR-visual (**a**, **d**) and TSR-auto(median) (**b**, **e**). Results for patients with stage II rectal cancer (*n* = 45) are shown for TSR-auto(median) only (**c**, **f**). Log-rank test *p*-values are shown in the graphs

Survival analysis generally showed a worse outcome for stroma-high patients compared to stroma-low patients (Fig. 3), independent of the method of TSR assessment used (visual versus automated). For TSR-visual, the 5-year survival rates for stroma-low versus stroma-high cases were 71.0% versus 58.8% for DSS and 65.6% versus 49.1% for DFS. For TSR-auto(50%), the 5-year survival rates for stroma-low versus stroma-high cases were 86.6% versus 60.7% for DSS and 76.8% versus 54.9% for DFS. For TSR-auto(median), the 5-year survival rates for stroma-low versus stroma-high cases, were 76.1% versus 58.4% for DSS and 70.0% versus 50.7% for DFS.

For TSR-visual, a significantly lower DSS was seen in the stroma-high group compared to the stroma-low group (*p* = 0.042), but not for DFS (*p* = 0.182). Similarly, for TSR-auto(50%) this difference was significant for DSS (*p* = 0.018), but not for DFS (*p* = 0.066). For TSR-auto(median), both DSS and DFS were found to be significantly lower in the stroma-high group compared to the stroma-low group (*p* = 0.007 and *p* = 0.021, respectively). After stratification for TNM stage, stroma-high was also found to be associated with worse survival in stage II rectal cancer patients (*n* = 45), but this result was only significant for TSR-auto(median) (DSS *p* = 0.003 and DFS *p* = 0.015).

Hazard ratios (HR) and 95% CIs were determined for both DSS and DFS (Tables 6 and 7). In univariate analysis, all methods for TSR assessment were found to be prognostic for DSS: TSR-visual HR = 1.83 (95% CI 1.01–3.30); TSR-auto(50%) HR = 2.71 (95% CI 1.14–6.40); and TSR-auto(median) HR = 2.31 (95% CI 1.24–4.30). For DFS, only TSR-auto(median) was found to be prognostic with HR = 1.96 (95% CI 1.10–3.51). After stratification for TNM stage, only TSR-auto(median) was found to be prognostic for stage II rectal cancer patients, both for DSS (univariate HR = 4.13 (95% CI 1.53–11.16)) and DFS (univariate HR = 3.05 (95% CI 1.19–7.81)).

**Table 6:** Uni- and multivariate Cox regression analysis for disease-specific survival

	Univariate		Multivariate					
			TSR-visual^a^		TSR-auto(50%)		TSR-auto(median)	
	HR	95% CI	HR	95% CI	HR	95% CI	HR	95% CI
Age^b^	1.01	0.98–1.04	1.01	0.98–1.04	1.00	0.98–1.03	1.01	0.98–1.04
Gender								
Female	1.00		1.00		1.00		1.00	
Male	1.16	0.61–2.17	1.64	0.83–3.24	1.67	0.83–3.34	1.62	0.83–3.18
T-status^c^								
pT1–pT2	1.00		1.00		1.00		1.00	
pT3–pT4	**4.52**	**1.91–10.72**	**3.75**	**1.56–9.03**	**4.80**	**1.96–11.75**	**4.48**	**1.84–10.91**
LN metastases^d^								
No	1.00		1.00		1.00		1.00	
Yes	**2.93**	**1.35–6.32**	1.64	0.53–5.08	1.43	0.45–4.57	1.26	0.42–3.76
Tumor grade^e^								
Well–Moderate	1.00		1.00		1.00		1.00	
Poor	**2.86**	**1.32–6.20**	**2.87**	**1.24–6.69**	2.29	0.96–5.47	**2.63**	**1.14–6.08**
Adjuvant therapy								
No	1.00		1.00		1.00		1.00	
Yes	**2.31**	**1.28–4.15**	1.28	0.39–4.17	1.25	0.37–4.22	1.42	0.44–4.54
TSR-visual^a^								
Stroma-low	1.00		1.00					
Stroma-high	**1.83**	**1.01–3.30**	1.76	0.93–3.34				
TSR-auto(50%)								
Stroma-low	1.00				1.00			
Stroma-high	**2.71**	**1.14–6.40**			**3.11**	**1.26–7.70**		
TSR-auto(median)								
Stroma-low	1.00						1.00	
Stroma-high	**2.31**	**1.24–4.30**					**2.48**	**1.29–4.78**

^a^Observer consensus

^b^Age was used as a continuous variable

^c^Due to low numbers, pT1 (*n* = 4) and pT2 cases were grouped together as well as pT3 and pT4 (*n* = 6) cases

^d^Lymph node metastases

^e^Due to low numbers, cases with well (*n* = 3) and moderately differentiated tumors were grouped together

Significant results (*p* > 0.05) are indicated in bold

**Table 7:** Uni- and multivariate Cox regression analysis for disease-free survival

	Univariate		Multivariate					
			TSR-visual^a^		TSR-auto(50%)		TSR-auto(median)	
	HR	95% CI	HR	95% CI	HR	95% CI	HR	95% CI
Age^b^	1.00	0.97–1.02	1.00	0.97–1.02	0.99	0.96–1.02	0.99	0.97–1.02
Gender								
Female	1.00		1.00		1.00		1.00	
Male	1.35	0.73–2.52	1.87	0.96–3.63	1.80	0.92–3.52	1.81	0.94–3.49
T-status^c^								
pT1–pT2	1.00		1.00		1.00		1.00	
pT3–pT4	**4.09**	**1.83–9.12**	**3.52**	**1.55–8.01**	**4.33**	**1.87–10.04**	**4.11**	**1.78–9.48**
LN metastases^d^								
No	1.00		1.00		1.00		1.00	
Yes	**2.43**	**1.38–4.28**	1.89	0.68–5.25	1.78	0.63–5.00	1.55	0.58–4.19
Tumor grade^e^								
Well–Moderate	1.00		1.00		1.00		1.00	
Poor	**2.24**	**1.05–4.80**	2.02	0.88–4.62	1.65	0.70–3.87	1.78	0.78–4.06
Adjuvant therapy								
No	1.00		1.00		1.00		1.00	
Yes	**2.22**	**1.26–3.89**	1.07	0.37–3.10	0.98	0.33–2.90	1.11	0.39–3.15
TSR-visual^a^								
Stroma-low	1.00		1.00					
Stroma-high	1.47	0.83–2.61	1.42	0.77–2.61				
TSR-auto(50%)								
Stroma-low	1.00				1.00			
Stroma-high	2.01	0.94–4.29			**2.39**	**1.07–5.38**		
TSR-auto(median)								
Stroma-low	1.00						1.00	
Stroma-high	**1.96**	**1.10–3.51**					**2.05**	**1.11–3.78**

^a^Observer consensus

^b^Age was used as a continuous variable

^c^Due to low numbers, pT1 (*n* = 4) and pT2 cases were grouped together as well as pT3 and pT4 (*n* = 6) cases

^d^Lymph node metastases

^e^Due to low numbers, cases with well (*n* = 3) and moderately differentiated tumors were grouped together

Significant results (*p* > 0.05) are indicated in bold

In multivariate analysis, automated TSR assessment was found to be prognostic independent of age, gender, pT-stage, lymph node status, tumor grade, and whether adjuvant therapy was given, both for DSS: TSR-auto(50%) HR = 3.11 (95% CI 1.26–7.70) and TSR-auto(median) HR = 2.48 (95% CI 1.29–4.78), and for DFS: TSR-auto(50%) (HR = 2.39 (95% CI 1.07–5.38)) and TSR-auto(median) (HR = 2.05 (95% CI 1.11–3.78)). TSR-visual was not found to serve as an independent prognostic factor.

## Discussion

For different cancer types, TSR has been shown to yield prognostic information. Visual assessment of TSR requires training, and may be difficult for cases close to the decision threshold of 50%. The present study shows that specifically for rectal adenocarcinoma the observer agreement is only moderate. Recent advances in slide scanning technology and machine learning have opened up new possibilities for computerized assessment of TSR. To the best of our knowledge, the present study shows for the first time that TSR can reliably be assessed by an automatic deep learning algorithm. The agreement of the automated system (using median cut-off) with the observer consensus (kappa = 0.521) was comparable to the inter-observer agreement (kappa = 0.578). The TSR assessed in this manner appeared to be a strong independent prognostic factor both for DSS and DFS in rectal adenocarcinoma. The prognostic value of the automated TSR was comparable to that assessed in consensus by two experienced observers for DSS in univariate analysis, but not in multivariate analysis. For DFS, only the automatically assessed TSR was significantly associated with outcome, both in univariate and multivariate analysis.

Interestingly, automated TSR (using the median as cut-off) showed prognostic value for TNM stage II patients. Clinically, this is a subgroup of patients for which post-operative treatment is still under debate and more research is needed [22, 23]. TSR can potentially help to direct this discussion and add information for a more personalized treatment of this patient category.

In a recent study, Scheer et al. [8] analyzed TSR on the same cohort of patients as used in the present study. However, rather than a hot-spot measure, the authors applied a scoring procedure in which an average TSR was assessed based on the entire tumor area in a slide. Also, they defined TSR as the carcinoma percentage (CP) and the estimated percentages were grouped using three categories (low-CP, intermediate-CP and high-CP). In univariate survival analysis, CP was found to be prognostic for DSS and DFS. With CP-high as baseline and after correction for age, grading, pathological T-stage, and adjuvant treatment, CP-intermediate was found to be correlated with worse DSS and DFS, however, this result was obtained only in the subset of lymph node metastasis negative cases (*n* = 94). In the present study, the prognostic value of TSR remained intact for the entire cohort of patients after correction for clinicopathological variables, including lymph node status. The most probable cause for this difference is the TSR scoring method. In the present study we decided to follow a more widely accepted scoring system, which appears to outperform methods where the overall tumor area is scored by averaging.

The results of our observer study indicate that TSR obtained by visual estimation serves as a prognostic factor of DSS (although not reaching statistical significance when correcting for other clinicopathological features), but not of DFS. Furthermore, only a moderate agreement was found between observers. These results are in contrast with previous studies [9, 10, 13] on TSR assessment on colon cancer. This discrepancy may be explained by the fact that compared to colon, the rectum bowel wall has a thicker muscle layer and in, some cases, it may be difficult to distinguish between stromal tissue and smooth muscle cells, especially with darker H&E-stained slides. Muscle tissue, which should be excluded from scoring, may therefore be interpreted as stromal tissue by one observer and not by the other. Furthermore, as shown in Fig. 2, most discrepancies (15/24 cases) are found around the cut-off point of 50%. Especially for these cases, computer-aided TSR assessment may be very useful.

For the automated method two different stroma cut-off values have been investigated in this study: the value used for the visual estimation (50%), and the median of measured TSR-auto values. We found comparable results for the two cut-offs, with a slightly higher hazard ratio for the 50% cut-off at the cost of a wider 95% confidence interval. However, since in general automated assessment of TSR yields higher stroma percentages than visual assessment, the use of a 50% cut-off for TSR-auto corresponded much less to TSR-visual compared to the use of the median cut-off (as is reflected in the kappa values). The optimal cut-off value for TSR-auto should be further investigated and validated in an independent cohort.

It is worth noting that one of the patient inclusion criteria for the cohort that was used in this study was the absence of neoadjuvant treatment. The reason for this design choice, originally made by Scheer et al. [8], was that both chemotherapy and radiotherapy modifies the tissue architecture and, as such, may hamper the assessment of TSR or its prognostic value. The proposed method can, therefore, aid clinicians in selecting the right treatment options for rectal cancer patients who did not receive preoperative (chemo)radiotherapy. Furthermore, given the fact that the colon and the rectum are parts of the same continuous organ and have a similar histological appearance, the presented deep learning algorithm has the potential to be successfully applied to the analysis of colon cancer as well.

The deep learning-based approach proposed in this work needs the position of a user-provided stroma hot-spot as input in order to assess TSR. After this manual input is provided, the proposed method can process the hot-spot area in the whole-slide image automatically. As such, human input is still required, making the method only semi-automatic. It is worth noting that in Ciompi et al. [20] a computer model similar to the one used in this work has shown a high performance at segmenting several tissue types in rectal cancer at the whole-slide image level, i.e., beyond the limited area of the selected hot-spot. As a consequence, this method has the potential to be used to assess TSR both at whole-tumor level and at whole-slide image level. Such an approach would overcome the need for a user-provided stroma hot-spot and, therefore, allow investigating TSR at very large scale via fully-automatic computation. Future work will be directed towards further automation of TSR assessment and validation in a large independent cohort.

Although, to the best of our knowledge, TSR assessment (visual or automated) has not yet been implemented in routine pathology diagnostics, it was recently reported [24] that the TNM Evaluation Committee (UICC) and the College of American Pathologists (CAP) have discussed TSR and acknowledged its potential for integration with the TNM staging system. To achieve this for colon cancers, we are currently investigating the reproducibility of (visual) TSR assessment in a large European multicenter study [25]. The results of the present study suggest that automated TSR can potentially be of significant aid to pathologists in routine diagnostics. However, validation of the proposed technology on a larger and independent data set is essential and, therefore, among our future research goals. The objectiveness of a deep learning-based method, which allows obtaining accurate and reproducible quantification of TSR, has the potential to pave the way to implementation of TSR in clinical practice.
